# Supplementary material for: P-TRAP: a Panicle Trait Phenotyping tool
Source: BMC Plant Biol. 2013 Aug 29;13:122. doi: 10.1186/1471-2229-13-122 (PMC3848748; doi:10.1186/1471-2229-13-122)
Supplement: Additional file 7 — P-TRAP source code. The Netbeans project that contains the source code of the application. [file 1471-2229-13-122-S7.zip › javadoc.zip!/javadoc/org-bioalg-imageprocessor/allclasses-frame.html]

All Classes (Image Processor)


# All Classes

- AImage
- AImage.CONNECTIVITY
- ColoredImage
- EDistanceMap
- HSBPixel
- ImageIO
- Kernels
- MathMorphology
- MathMorphology.MorphingType
- RGBPixel
